# Examiner training and calibration for simulated clinical examinations: A scoping review

**DOI:** 10.1111/medu.70253

**Published:** 2026-06-25

**Authors:** Harish Thampy, Niamh Callanan, Arslan Ahmed, Sophia Taromsari

**Affiliations:** ^1^ Faculty of Biology, Medicine and Health University of Manchester Manchester UK; ^2^ Clinical Teaching Fellow Manchester University NHS Foundation Trust Manchester UK

## Abstract

**Introduction:**

Examiner training and calibration are widely recommended to improve scoring consistency and defensibility in simulation‐based observed clinical competency assessments (SOCCAs), yet the empirical evidence has not yet been comprehensively explored. This scoping review maps and describes studies evaluating general examiner training and calibration interventions in SOCCAs.

**Methods:**

A scoping review was conducted of peer‐reviewed studies that reported empirical evaluation or descriptive outcomes of examiner training and/or calibration in any SOCCA format (including OSCEs and OSLERs) across health profession disciplines and training stages. Data were charted on intervention characteristics, study design, outcome measures and reported effects. Given study heterogeneity, findings were synthesised descriptively.

**Results:**

Twenty studies met inclusion criteria. Seven described general examiner training, although intervention reporting and evaluation were often insufficient to judge effectiveness. Two broad calibration approaches were identified: (i) general calibration aimed at aligning examiners' internal performance standards and (ii) case‐orientation calibration tailored to specific stations subsequently assessed. Most calibration interventions used frame‐of‐reference approaches, commonly involving video‐recorded benchmark performances with facilitated discussion. Effects on scoring outcomes were inconsistent: some studies reported modest improvements in scoring reliability or accuracy, whereas others showed minimal change or increased examiner stringency. Only one study assessed outcomes beyond scoring metrics to study examiner behaviours.

**Discussion:**

Despite widespread endorsement, the evidence base for examiner training and calibration in SOCCAs remains limited and inconsistent. Where benefits were observed, they appeared most evident for borderline/uncertain performances, suggesting calibration may be most useful near decision thresholds. Additionally, this review highlights an unresolved tension in calibration aims, whether to promote score convergence, shared reasoning or both. Future work should specify intended mechanisms, address contextual influences on judgement and extend evaluation to impact on assessor behaviour. In the absence of stronger evidence, routine implementation risks being driven by expectation and convention rather than a sufficiently robust empirical rationale.

## Abstract 2

Examiner training & calibration are typically mandated in high‐stakes clinical exams but does the evidence support this? Our scoping review in Medical Education maps the evidence in this area. #MedEd

## 1 INTRODUCTION

Clinical skill assessments, such as the Objective Structured Clinical Examination (OSCE) and the Objective Structured Long Examination Record (OSLER), are widely used across undergraduate and postgraduate health professions education.^1^, ^2^ These assessments evaluate performance at the ‘shows how’ level of Miller's pyramid, requiring candidates to demonstrate the application of clinical knowledge, skills and professional behaviours within time‐limited, observed clinical encounters.^3^ Although the format of these assessments varies, they share key features relevant to examiner judgement, including structured tasks, standardised marking criteria and the use of constructed assessment encounters that are distinct from routine clinical care. In this review, such assessments are grouped under the term *simulation‐based observed clinical competency assessments* (SOCCAs). The term ‘simulated’ is used here to denote the assessment context rather than the use of simulated patients, such that OSCEs, OSLERs and related formats are considered simulated assessments even when real patients are involved.

Although often described as objective, SOCCAs necessarily involve examiner judgement when scoring observed performance, thereby introducing potential sources of variability. Indeed, examiner‐related factors account for a sizeable proportion of score variance within SOCCAs,^4^, ^5^ and fairness and comparability are compromised when examiners are not adequately trained for their role.^6^ Examiner variability, manifesting as stringency, leniency or differing interpretations of performance standards, therefore threatens the validity of these clinical assessments.^5^, ^7^, ^8^, ^9^ In response, rater training has been proposed to mitigate assessor variability and has been studied across sectors, including the military, industry, and health professions education.

Four principal rater training approaches have been described: rater error training, performance dimension training, frame‐of‐reference training and behavioural observation training.^10^, ^11^, ^12^ Rater error training focuses on recognising and reducing common judgement errors; performance dimension training clarifies constructs and boundaries of performance domains; frame of reference training extends performance dimension training by aligning how performance quality is judged within each dimension, often through rating exemplars and structured discussion; and behavioural observation training targets observation and recall of performance‐relevant behaviours.

Within health professions education, rater training has been investigated predominantly in the context of workplace‐based assessments (WBAs), with mixed findings. Some interventions have improved scoring accuracy, particularly when intensive and multi‐component,^13^ while others have shown no sustained impact or have increased examiner severity.^14^, ^15^ Furthermore, more experienced WBA examiners may be less influenced by training, especially when reference standards conflict with established schemas of competence.^16^, ^17^ However, SOCCAs differ from WBAs in important structural and epistemic ways as they are episodic, highly standardised, time‐limited assessments in which examiner judgements are typically made under constrained conditions and contribute to high‐stakes decisions. Consequently, findings from WBA‐focused rater training literature cannot be assumed to transfer directly to simulated assessment contexts, supporting the need to examine the SOCCA‐specific evidence base in its own right.

Regulatory and professional guidance frequently endorses examiner training and calibration to improve fairness, reliability and validity in SOCCAs.^18^, ^19^, ^20^ In high‐stakes settings, examiner judgements in SOCCAs carry significant consequences for learners and institutions, including progression and licensure decisions, exposure to complaints and appeals, and challenges to the defensibility of assessment outcomes. The effectiveness of examiner training and calibration practices therefore has implications not only for educational quality but also for institutional credibility and public trust. In the UK, the introduction of the Medical Licensing Assessment has further increased scrutiny of clinical assessment processes, requiring medical schools to evidence approaches to examiner calibration within clinical skills assessments.^21^

Despite strong regulatory and institutional expectations for examiner consistency, the evidence base underpinning examiner training and calibration in SOCCAs has not been systematically mapped to examine the range of approaches, their design and reported outcomes. Approaches to examiner training and calibration may draw on differing conceptions of judgement, including whether variability is treated as a problem of measurement to be reduced through standardisation and score convergence, or as an interpretive process shaped by context and shared professional understanding.^7^ These distinctions have implications for how such interventions are conceptualised and evaluated, and provide further rationale for examining the SOCCA‐specific literature.

This review seeks to address this gap by systematically mapping the literature on examiner training and calibration in SOCCAs across health professions education. We adopt the term *examiner training* to refer to *general* structured activities designed to prepare assessors for their role, including orientation to the examination format, logistics and marking procedures. *Examiner calibration* is used to refer to *specific* interventions that promote alignment in how examiners apply scoring criteria, whether during training exercises or live assessments, to improve consistency and accuracy of examiner scoring.

## 2 METHODS

A scoping review approach was adopted following the five‐stage Arksey and O'Malley methodological framework.^22^ Scoping reviews are useful for examining a broad spectrum of literature to map key concepts, evidence types and research gaps within a given field. They are particularly appropriate in areas where rigorous or consistent empirical evidence is limited and thus incorporate studies of varied design and methodological quality.^23^ Given the anticipated diversity of intervention designs, theoretical framings and outcome measures within the examiner training and calibration literature, a scoping review was considered more appropriate than theory‐testing or explanatory synthesis approaches. Under these conditions, a scoping review methodology enables systematic mapping of the characteristics and conceptual assumptions of the evidence base, while identifying areas where more theory‐driven synthesis would be premature. Consistent with many scoping reviews, the optional consultation stage described by Arksey and O'Malley was not undertaken. The purpose of this review was to map and characterise the existing empirical literature on examiner training and calibration in SOCCAs, rather than to co‐produce recommendations or examine implementation with stakeholders; stakeholder consultation was therefore considered beyond the scope of the review. The Preferred Reporting Items for Systematic Reviews and Meta‐Analyses extension for Scoping Reviews (PRISMA‐ScR) framework was employed to guide and structure the reporting of this review.^24^

### 2.1 Stage 1: Identifying the research question

To address our aim, this review aimed to answer the following research questions:

1. What strategies have been reported to prepare examiners for their role in SOCCAs?
2. How do different calibration modalities, including rater‐training approach, format, and timing, affect examiner scoring accuracy and behaviours?

### 2.2 Stage 2: Identifying relevant studies

The review included peer‐reviewed published literature, including empirical evaluations and descriptive reports where interventions and outcomes were reported. With the assistance of a librarian, four databases (PubMed, ERIC, Web of Science and Scopus), were systematically searched in November 2024, with an update conducted in January 2026 to capture newly published studies. The search terms used in each database are detailed in Table 1. Studies were included if they described and evaluated interventions related to general examiner training and/or examiner calibration in any SOCCA format, including OSCEs and OSLERs. Papers from all health profession disciplines and from both undergraduate and postgraduate contexts were eligible for inclusion. The full inclusion and exclusion criteria are presented in Table 2.

**TABLE 1:** Database‐specific search strings.

Database	Search strategy
PubMed	1:((‘clinical exam*’[Title] OR ‘medical exam*’[Title] OR ‘nursing exam*’[Title] OR ‘dentistry exam*’[Title] OR ‘HPE clinical exam*’[Title] OR ‘Clinical Competency Assessment*’[Title] OR OSCE*[Title] OR ‘Objective structured clinical exam*’[Title] OR ‘OSLER exam*’[Title] OR ‘summative clinical exam*’[Title] OR ‘postgraduate clinical exam*’[Title] OR ‘undergraduate clinical exam*’[Title] OR ‘simulated exam*’[Title] OR ‘simulated assessment*’[Title] OR ‘clinical skills exam*’[Title])) 2:(standardi*ation[Title/Abstract] OR standardi*ed[Title/Abstract] OR calibration[Title/Abstract] OR reliability[Title/Abstract] OR validity[Title/Abstract] OR consistency[Title/Abstract] OR best practice*[Title/Abstract]) 3:((examiner*[Title/Abstract] OR assessor*[Title/Abstract] OR interrater*[Title/Abstract] OR inter‐rater*[Title/Abstract]) AND (train*[Title/Abstract] OR prepar*[Title/Abstract])) 4:#2 OR #3 5:#1 AND #4
ERIC	1: TI (((‘clinical exam*’ or ‘medical exam*’ or ‘nursing exam*’ or ‘dentistry exam*’ or ‘HPE clinical exam*’ or ‘ Clinical Competency Assessment*’ or OSCE* or ‘Objective structured clinical exam*’ or ‘OSLER exam*’ or ‘summative clinical exam*’ or ‘postgraduate clinical exam*’ or ‘undergraduate clinical exam*’ or ‘simulated exam*’ or ‘simulated assessment*’ or ‘clinical skills exam*’))) 2:TI ( (examiner* or assessor* or Interrater* or inter‐rater*) AND (train* or prepar*) ) OR AB ((examiner* or assessor* or Interrater* or inter‐rater*) AND (train* or prepar*)) 3:TI ((standardisation or standardised or calibrat* or reliability or validity or consistency or best practice*) ) OR AB ((standardi?ation or standardi?ed or calibrat* or reliability or validity or consistency or best practice*)) 4:2 OR 3 5:1 AND 4
Web of Science	1: TI = ((‘clinical exam*’ or ‘medical exam*’ or ‘nursing exam*’ or ‘dentistry exam*’ or ‘HPE clinical exam*’ or ‘ Clinical Competency Assessment*’ or OSCE* or ‘Objective structured clinical exam*’ or ‘OSLER exam*’ or ‘summative clinical exam*’ or ‘postgraduate clinical exam*’ or ‘undergraduate clinical exam*’ or ‘simulated exam*’ or ‘simulated assessment*’ or ‘clinical skills exam*’)) 2: (TI=(((examiner* or assessor* or Interrater* or inter‐rater*) AND (train* or prepar*)))) OR AB=(((examiner* or assessor* or Interrater* or inter‐rater*) AND (train* or prepar*))) 3: (TI = ((standardi?ation or standardi?ed or calibrat* or reliability or validity or consistency or best practice*))) OR AB = ((standardi?ation or standardi?ed or calibrat* or reliability or validity or consistency or best practice*)) 4: #3 OR #2 5: #4 AND #1
Scopus	(TITLE (( ‘clinical exam*’ OR ‘medical exam*’ OR ‘nursing exam*’ OR ‘dentistry exam*’ OR ‘HPE clinical exam*’ OR ‘Clinical Competency Assessment*’ OR OSCE* OR ‘Objective structured clinical exam*’ OR ‘OSLER exam*’ OR ‘summative clinical exam*’ OR ‘postgraduate clinical exam*’ OR ‘undergraduate clinical exam*’ OR ‘simulated exam*’ OR ‘simulated assessment*’ OR ‘clinical skills exam*’)) ) AND ( ( ( TITLE‐ABS ( ( examiner* OR assessor* OR interrater* OR inter‐rater*)) ) AND ( TITLE‐ABS ( ( train* OR prepar*)) ) ) OR (TITLE‐ABS (((standardi?ation OR standardi?ed OR calibrat* OR reliability OR validity OR consistency OR ‘best practice*’)) ) ))

**TABLE 2:** Inclusion and exclusion criteria.

Inclusion criteria	Exclusion criteria
Available in the English language	No available English language version
Involving simulation‐based observed clinical competency assessments (SOCCAs), including OSCE/OSLER formats	Involving examination methods outside of the context of simulation‐based observed clinical competency assessments (SOCCAs)
Specific to the examination of clinical skills within any health‐care discipline	Relating to examination methods unrelated to clinical skills or conducted out with health‐care disciplines
Relating to the examination of undergraduate and/or postgraduate students	
Relating to the preparation or training of examiners for their role	

### 2.3 Stage 3: Study selection

The article selection process is summarised in the PRISMA flow diagram (Figure 1). Screening was conducted by three reviewers through title, abstract and full‐text stages, with each record independently reviewed by two reviewers at each stage. To support consistency in the application of the inclusion and exclusion criteria, all three reviewers jointly screened an initial subset of 10 papers and discussed discrepancies to calibrate decision‐making. Following this, dual screening was continued across all stages. Formal inter‐rater reliability statistics were not calculated, as the purpose of calibration was to promote consistent application of eligibility criteria rather than to quantify agreement, consistent with scoping review methodology. Following identification of the core set of eligible studies, forward and backward citation tracking (“snowballing”) was conducted to locate additional relevant articles aligned with the review's research questions.

**FIGURE 1:**
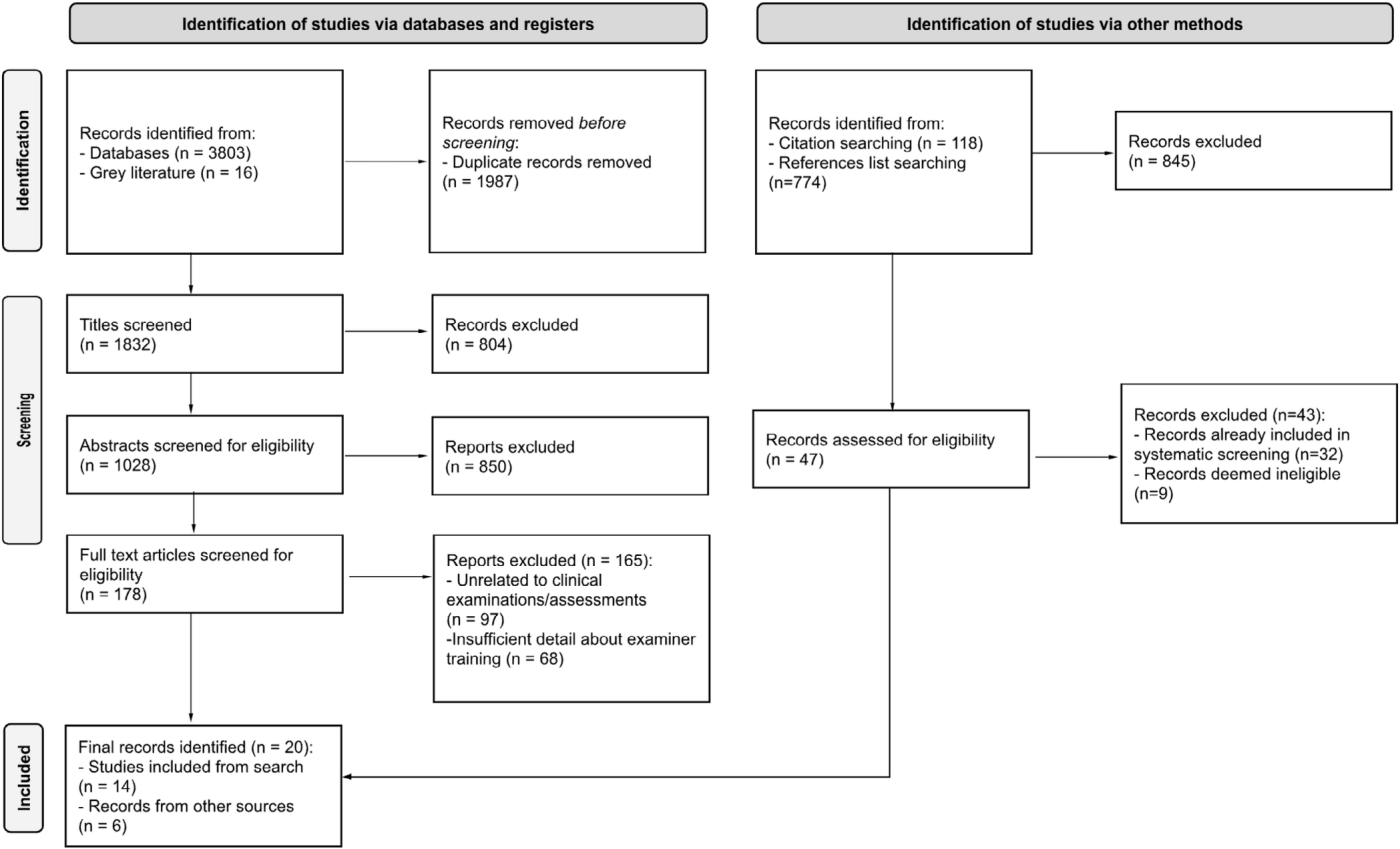
PRISMA flow diagram of study selection. PRISMA, Preferred Reporting Items for Systematic Reviews and Meta‐Analyses.

### 2.4 Stage 4: Charting the data

Each reviewer completed a structured data extraction form in Microsoft Excel designed to capture key characteristics of each paper. The data extraction framework was designed to align with the review aims of describing the characteristics, theoretical orientation, and reported effects of examiner training and calibration in SOCCAs. Extracted items therefore captured intervention design and format, training or calibration approach, timing relative to assessment and reported outcomes, permitting descriptive comparison across heterogeneous study designs and supporting interpretation of findings in relation to differing assumptions about examiner judgement. Each included paper was independently charted by two authors. Discrepancies in extracted data or categorisation were resolved through discussion, with the supervising author acting as one of the independent extractors and reviewing the final agreed charting prior to synthesis. Where intervention descriptions allowed, reviewers categorised interventions by rater‐training type (rater error training, performance dimension training, frame‐of‐reference training and behavioural observation training) and categorised the evaluation of each study using Kirkpatrick's four‐level model of training evaluation.^25^ Our categorisation of the Kirkpatrick levels is provided in Table 3. Given the heterogeneity of study designs and outcome timing, application of Kirkpatrick levels required interpretive judgement, and some studies did not align cleanly with a single level.

**TABLE 3:** Kirkpatrick level definitions.

Level	Applied definition
Reaction (1)	Evaluating examiner reactions and perceptions such as reported usefulness/satisfaction of the intervention
Learning (2)	Evaluating examiner learning related to scoring (e.g. improved accuracy against benchmarks, improved understanding of criteria), measured immediately after training
Behaviour (3)	Evaluating short‐term changes in examiner behaviour following the intervention
Results (4)	Evaluating sustained examiner scoring/behaviour outcomes in subsequent live examinations (beyond immediate post‐training testing)

### 2.5 Stage 5: Summarising and reporting findings

The charted data were synthesised using a descriptive thematic approach across examiner training and calibration categories. Synthesis focused on identifying recurring patterns in intervention characteristics, implementation approaches, evaluation strategies and reported outcomes, rather than on aggregating effect estimates. Given the heterogeneity of study designs and reporting, interpretive judgements were required when grouping interventions and outcomes; these were discussed among the reviewing authors to promote consistency and transparency. Reflexivity was addressed through iterative discussion of emerging interpretations and by explicitly linking synthesis decisions to the data extracted from individual studies.

## 3 RESULTS

Twenty studies met the inclusion criteria and were included in the final synthesis and are summarised in Table 4. Across the dataset, substantial heterogeneity was observed in intervention design, delivery modality, duration and outcome measures as described in the following themes. Reported outcomes included participant reactions (Kirkpatrick Level 1), changes in scoring accuracy (as compared with expert panel ratings) and changes in scoring reliability (as compared with other examiners). Given the near‐exclusive focus of the included studies on frame‐of‐reference approaches, the available evidence did not permit meaningful comparison between alternative calibration modalities.

**TABLE 4:** Results table presenting the final set of included articles and a summary of their findings.

Title, author and year of publication (sorted by year of publication)	Health‐care Discipline	Intervention type	Timing between intervention and actual scoring (for case‐orientation calibration only)	Intervention details	Key outcomes	Kirkpatrick level
The selection and training of examiners for clinical examinations (Newble et al, 1980)	Medicine	Case‐orientation calibration ‐ FOR + PDT	2 months	Eighteen examiners scored video‐recorded neurological lower limb examinations using structured checklists. Following initial ratings (run one), three subgroups were created (a) no further training, (b) limited training (30‐min session covering criteria for an adequate performance and discussion of the marking rubric) and (c) extensive training (as per limited plus 2 further hours for group analysis and discussion of a video‐simulated performance). After 2 months, examiners re‐rated the same videos (run two).	Training did not significantly improve inter‐rater reliability.	2
Improving OSCE examiner skills in a Malaysian setting (Tan and Azila 2007)	Medicine	General calibration ‐ FOR	Unspecified	Thirty‐six examiners attended a workshop in which they scored students in a four‐station– simulated OSCE. Use of a 2‐way mirror allowed evaluation of examiner behaviour and scoring followed by discussion of scores. Examiners were then observed again in a subsequent OSCE although timing between the training and the later OSCE is not provided.	Trained examiners were reported to be more consistent in scoring for subsequent OSCEs and less likely to prompt students compared to untrained examiners though no numerical data provided.	3
Assessor training: its effects on criterion‐based assessment in a medical context (Pell et al 2008)	Medicine	Training + general calibration (FOR)		Examiners undertook a half‐day course with initial general training followed by independent marking of live and videoed stations with group discussion to achieve consensus. Impact of training explored using data from a subsequent 12‐station summative OSCE.	Untrained assessors awarded higher marks than those who had been trained.	2
Assessment of patient communication skills during OSCE: examining effectiveness of a training programme in minimising inter‐grader variability (Schwartzman et al, 2011)	Pharmacy	Training + case‐orientation calibration ‐ FOR	Immediate	A 45‐minute on the day OSCE training attended by both examiners and SPs (who both scored each OSCE). Training included an orientation to the marking rubric followed by case‐orientation discussion using video examples of the allocated OSCE station examiners, and SPs were due to assess.	Inter‐rater (between SPs and examiners) variation pre‐intervention and post‐intervention were both statistically significant, although the difference was statistically lower following the intervention.	2
Awarding global grades in OSCEs: evaluation of a novel eLearning resource for OSCE examiners (Gormley et al, 2012)	Medicine	General calibration ‐ FOR		Evaluation of an e‐learning training package that focused on how to award a global grade followed by a scoring activity of at least three simulated OSCE performance videos. 200 examiners completed the training and were able to see their scores as compared with previous users.	86.5% either strongly agreed or agreed that the resource was useful in their ability to award global grades in OSCEs. There was statistically significant improvement in confidence awarding grades after using the training package.	1
Effectiveness of rater consensus training in objective structured clinical examination using Kolb's experiential learning (Chu et al, 2012)	Medicine	Case‐orientation calibration ‐ FOR		Raters completed an e‐learning course before attending a half‐day workshop which occurred 1 day before a summative OSCE. Insufficient details provided, but description suggests raters scored and then discussed an unspecified number of performances of unspecified performance levels. Participants then took part the following day in an eight‐station OSCE.	Moderate to high inter‐rater reliability was seen for all stations in the subsequent summative OSCE.	2
Twelve tips for designing and implementing a structured rater training in OSCEs (Preusche et al., 2012)	Various	Case‐orientation calibration ‐ FOR+RET+PDT	Unspecified	Examiners attended a 3‐h workshop 1 week prior to OSCE specific to the station they were going to examine (either history taking, examination or procedural skills). Training included discussion on rater error, and participants were asked to consider what would be required of the minimally competent candidate. Examiners then watched three videos with group discussion of scoring (though which levels of performance is not specified). Onward OSCE scoring outcomes compared for trained and untrained examiners.	Trained examiners showed greater variation in their scores and made more fail decisions than untrained raters.	2
Correlation of rater training and reliability in performance assessment: Experience in a school of dentistry (Lin et al., 2013).	Dentistry	Case‐orientation calibration ‐ FOR	1 month	Nine raters scored video‐recorded performances of two students doing an 8‐min OSCE station of a dental procedure. The raters then took part in once‐a‐week rater assessment programme over a month period, including role play, rating practice and discussion. After this 1‐month programme, the nine raters re‐scored the same two videos again.	Inter‐rater reliability was already high pre‐training for both videos though did statistically increase as a result of training.	2
Effects of a rater training on rating accuracy in a physical examination skills assessment (Weitz et al., 2014)	Medicine	Case‐orientation calibration ‐ FOR	4 days	Twenty‐one physician raters recruited to score 242 year 3 medical students who were videoed undertaking a physical examination OSCE station. Prior to scoring, the raters were split into two groups: (1) untrained and (2) trained using a 90‐min calibration workshop 4 days before the OSCE scoring exercise in which they watched and scored four recorded videos of differing performances followed by group discussion. Raters then scored a series of videos for the full cohort of students and their scores compared against a target score as determined by an expert panel.	Rater training did not have a measurable influence on accuracy. Trained raters were more stringent than untrained raters.	2
Key challenges for implementing a Canadian‐based objective structured clinical examination (OSCE) in a Middle Eastern context (Wilby and Diab, 2016)	Pharmacy	Training + general calibration (FOR)		Descriptive study comparing two methods used to calibrate examiners. 1: a 4‐h training session 1 month before the OSCE combined with a standardisation ‘dry run’ on the day of the OSCE. 2: assessors engaged in a formative OSCE for scoring familiarisation supplemented with large‐group discussion following role‐plays portraying different levels of performance. In the second approach, global assessment tools were also simplified to improve scoring consistency.	Inter‐rater reliability improved with the second approach for both checklist and global rating rubrics.	2
A pilot study of marking accuracy and mental workload as measure of OSCE examiner performance (Byrne et al., 2016)	Medicine	Training + general calibration ‐ FOR		Eighty participants attended an hour‐long slide‐based presentation explaining the OSCE process and marking. Each examiner then immediately completed four cycles of simulated marking practice (including varying performances) using four video‐recorded performances (one borderline and one excellent performance each for two different stations) with peer discussion after each video. Scores were compared with a pre‐agreed expert panel consensus score.	Training was not associated with an improvement in accuracy although no comparison was made to a control group. Accuracy improved after exposure to the same scenario and accuracy was greater when marking an excellent performance compared to a borderline performance.	2
Taking OSCE examiner training on the road: reaching the masses (Reid et al., 2016)	Medicine	Training + general calibration ‐ FOR		A 1‐h lecture covered OSCE station design, examiner roles and expected behaviours to 171 participants. It included scoring a single 9‐min mock OSCE station video (portraying a satisfactory performance) using a checklist, followed by discussion. Delivered at six grand rounds across five teaching hospitals. Examiner scores were compared to the ‘target’ score.	Eighty percent of participants scored the mock OSCE within a narrow range close to the ideal score. No significant differences in scoring were found by gender or professional role.	2
Effect of rater training on the reliability of technical skill assessments: a randomised controlled trial (Robertson et al, 2018)	Medicine	Case‐orientation calibration ‐ FOR	Immediate	Raters were randomly assigned to video‐based training (using a single video level of performance not specified) or no training. Participants then rated 10 blinded videos of trainees performing surgical suturing skills. Inter‐rater reliability assessed for each group.	Inter‐rater reliability was higher for the calibrated cohort versus the non‐trained group, although this was not statistically significant.	2
Training OSCE examiners: minimal effort with far‐reaching results (Schuttpelz‐Brauns et al., 2019)	Medicine	Training including RET + case‐orientation calibration (FOR)	Unspecified	Development of a three‐step training strategy for 165 participant examiners. (1) 40‐min interactive online course covering role of examiners, OSCE process and biases. (2) On the day station‐specific video review with peer discussion (although no detail given regarding number of videos and what levels of performance). (3) Trainers observed the participant examiners during a subsequent OSCE and provided structured feedback. Participants evaluated the effect of the training on knowledge and relevance, and their evaluation scores were compared with a control group who received only organisational material.	Creating the training materials and videos were initially time and resource intensive, although these could subsequently be re‐used. The intervention cohort reported statistically higher increases in scores for perceived knowledge and relevance than the control group.	1
Evaluation of a new e‐learning resource for calibrating OSCE examiners on the use of rating scales (Moreno‐López and Sinclair, 2020)	Dentistry	Case‐orientation calibration ‐ FOR		Online e‐learning package was developed for three stations. Two videos were produced per station to depict an unsatisfactory and an excellent candidate. Fifteen participants were then required to mark the six videos which was compared with a target score. Comparison of examiner mean scores were made to a ‘target’ score.	Examiner scoring was similar to target scores for the two consultation stations, although not for a procedural skill which the authors suggest may be attributable to camera placement issues. Examiners were positive in their feedback about the approach used.	2
Rater training for standardised assessment of Objective Structured Clinical Examinations in rural Tanzania (Sigalet et al., 2020)	Medicine, midwifery and nursing	Case‐orientation Calibration ‐ FOR	Immediate	Six participant raters were trained over 3 days where three levels of proficiency (poor, acceptable, excellent) for three stations were role‐modelled by research team members for mock scoring exercises. Raters then scored 42 role‐played scenarios for these three stations independently that were intended to depict one of the three levels of proficiency. Scores were collated and areas of disagreement explored.	Moderate inter‐rater agreement seen for the ‘poor’ and ‘acceptable’ level performances across the three stations. Raters were more accurate in identifying ‘poor’ and ‘excellent’ compared with average and often identified excellent proficiency level scenarios as average.	2
Effects of online frame‐of‐reference training on assessment accuracy in the objective structured clinical examination for physical therapy students (Watari et al., 2023)	Physiotherapy	Case‐orientation calibration ‐ FOR	1 month	Thirty‐six physical therapists were split into a control group and trained group. Both groups initially watched and scored three video performances of differing levels of a physical examination. The trained group then took part in two 90‐min online discussions based on rewatching the pre‐assessment videos. The control group only received an explanation of the rubric assessment chart. Following the intervention, both groups scored another three videos of the same physical examination, and this was conducted a third time 1‐month after the intervention to explore durability of effect.	Accuracy of scoring (as compared with an agreed target) was statistically higher at both the post‐ and 1 month post ‐ assessments for the trained group than control. However, training was not found to be effective for raters demonstrating high assessment accuracy before training.	2
Raters and examinees training for objective structured clinical examination: comparing the effectiveness of three instructional methodologies (Guerrero et al., 2024)	Nursing	Training	Unspecified	Eighteen raters and 54 examinees were equally divided into three groups of training methodologies: (1) a 70‐min online discussion and Q&A, (2) 80‐min in‐person lecture with Q&A, and three‐simulation exercise involving pre‐briefing, 108‐min mock OSCE scoring and then debriefing. Content of the first two groups was general training related. Insufficient detail provided on the simulation intervention. Participants then participated in a mock OSCE immediately after involving six procedural skill stations. Rater agreement for each cohort determined. Participant experience was evaluated through surveys and self‐assessments.	Simulation‐trained raters demonstrated the highest inter‐rater agreement, although even this was ‘slight’. Online‐trained raters showed the lowest agreement. Simulation‐trained raters reported higher perceived benefit than online and lecture cohorts.	2
Understanding contexts and mechanisms through which video‐based benchmarking promotes alignment of examiners' scoring in objective structured clinical exams (Edwards et al., 2025)	Medicine	Case‐orientation calibration ‐ FOR	Immediate	Sixteen participant examiners watched one of two possible benchmark videos (portraying either a ‘borderline’ or ‘good’ performance). Examiner were provided with benchmarking information (agreed scores and justifications) for their video either before or after observation. Examiners then scored a final video of a performance on the same task. Qualitative data collected through semi‐structured interviews.	Examiner expectations remained uncertain until viewing performance examples. The benchmarking exercise prompted reflection, with dissonance when their scores diverged from the expected score. How examiners engaged with the videos and feedback influenced whether they were willing to adjust to or dismiss the expected score. Video‐based benchmarking was reported to enhance preparedness, although no quantitative outcomes on effect on scoring was gathered.	1
Determining the influence of video‐based benchmarking (VBB) on examiner variability in Objective Structured Clinical Exams (OSCE). (Yeates et al. 2026)	Medicine	Case‐orientation calibration ‐ FOR	Immediate	Multicentre (12 medical schools and 171 assessors) stratified randomised controlled trial exploring the role of VBB versus a control intervention on subsequent OSCE station scoring (for a station on asthma management consultation). Intervention group watched two videos depicting an Excellent and Borderline performance followed by explanatory benchmarking information and group discussion (total 35 min) and then scored blind two target performances (scripted to Borderline and Good levels). Control group scored the two target performances without having participated in the calibration.	Primary endpoint: VBB did not impact on overall accuracy of examiner scoring. Secondary endpoints: VBB improved scoring accuracy for Borderline performances and reduced the observed variability for outlying examiners	2

Abbreviations: RET = rater error training; PDT = performance dimension training; FOR = frame‐of‐reference training; BOT = behavioural observation training.

### 3.1 General training interventions

Seven studies included some description of general orientation or induction programmes designed to prepare examiners for their role.^26^, ^27^, ^28^, ^29^, ^30^, ^31^, ^32^ Interventions typically included an overview of assessment principles, explanation of scoring rubrics, clarification of examiner roles and expected behaviours, and discussion of potential sources of bias, sometimes incorporating elements of rater error training. Short, structured interventions were associated with improved scoring accuracy,^31^ whereas less structured courses, or those focused primarily on exam logistics, were perceived as less useful.^29^ Across these studies, details about the training's pedagogy, duration and theoretical basis were largely lacking, with evaluation either minimal or limited to participant reactions (Kirkpatrick Level 1). Additionally, none of the included studies examined the role of refresher or update training, despite such training commonly being mandated across institutions for ongoing examiners.

### 3.2 Calibration interventions

This review identifies two distinct calibration approaches. General calibration was typically incorporated within examiner induction or broader training programmes and commonly employed scoring exercises to align examiners' internal performance standards with an aim to reduce idiosyncrasies. Case‐orientation calibration, by contrast, used similar approaches but was tailored to the specific station(s) that examiners would subsequently assess.

#### 3.2.1 General calibration interventions

Six studies reported general calibration activities.^27^, ^30^, ^31^, ^32^, ^33^, ^34^ All used frame‐of‐reference elements, via video or live role play, in which examiners scored performances, compared their judgements with peers or reference standards, and engaged in facilitated discussions to explore discrepancies and develop shared conceptualisations of performance. Schüttpelz–Brauns et al. noted the resource‐intensive nature of developing high‐quality video exemplars, although they acknowledged their reusability for future training.^29^

Evidence for effectiveness was mixed. Tan and Azila reported subsequent improved examiner reliability and fewer prompting behaviours in assessments that took place following initial general calibration, although no quantitative data were provided.^34^ Pell et al. found that calibrated examiners awarded lower scores and fewer passes than non‐calibrated examiners, suggesting increased stringency; however, accuracy against an agreed benchmark was not examined.^27^ While evidence of objective impact on examiner performance was limited, participation in general calibration exercises was reported to increase examiners' scoring confidence.^29^, ^33^

#### 3.2.2 Case‐orientation calibration

Thirteen papers investigated case‐orientation calibration interventions with exemplars and discussion specific to the station(s) for which examiners were due to score in an upcoming SOCCA.^26^, ^29^, ^35^, ^36^, ^37^, ^38^, ^39^, ^40^, ^41^, ^42^, ^43^, ^44^, ^45^ This was primarily delivered through frame‐of‐reference methods using video‐based exemplars in all but one study, which instead used in‐person role play.^36^ Some interventions incorporated additional rater training elements, such as rater error training^29^ or discussion of minimum competence thresholds in line with performance dimension training,^38^ although insufficient detail was provided to assess their independent contribution.

Several studies reported improvements in subsequent examiner scoring reliability and accuracy following frame‐of‐reference based case‐orientation calibration.^26^, ^37^, ^42^, ^43^ However, approaches varied in the number and range of exemplars, performance levels represented, and whether examiners rated exemplars blind to intended standards. Reporting was often insufficiently detailed to permit meaningful comparison of these design features or attribution of effects to particular components.

In contrast, other studies reported limited or no benefit to case orientation. Newble et al. observed no improvement in inter‐rater reliability following brief performance dimension training combined with extensive video‐based frame‐of‐reference training, although scoring occurred 2 months after the intervention, raising the possibility that any earlier effects may have attenuated.^39^ As with general calibration, case‐orientation calibration may also be associated with increased examiner stringency. Increased stringency was also reported following a 3‐h station‐specific calibration 1 week before an OSCE^44^ and after an extensive 3‐day programme in which ‘excellent’ performances were frequently downgraded to ‘average’.^36^

Edwards and colleagues aimed to examine *how* and *why* frame‐of‐reference‐based case orientation calibration works by exploring examiners' experiences of what they termed video‐based benchmarking (VBB), a frame‐of‐reference intervention in which examiners score station‐specific exemplars and then compare their ratings with an expert benchmark.^41^ The exercise reportedly enhanced preparedness and highlighted discrepancies between examiners' scores and expected standards. Examiners differed in whether they realigned or dismissed the benchmark, particularly when justifications were unconvincing or engagement was low. The authors also noted reduced cognitive engagement when examiners viewed performances with prior knowledge of the panel score. This work was expanded in a multicentre stratified randomised controlled trial across 12 medical schools (171 assessors) comparing VBB (two benchmarking videos) with control during subsequent OSCE scoring.^45^ VBB had no significant effect on overall examiner variability but improved scoring accuracy for borderline performances and reduced variability among outlying examiners.

### 3.3 Delivery modalities

#### 3.3.1 Online synchronous delivery

Two studies reported online synchronous delivery as part of training/calibration. Guerrero et al. found that online‐trained examiners demonstrated the lowest inter‐rater reliability in subsequent mock OSCE marking compared with in‐person lecture‐based and simulation‐based cohorts.^28^ In contrast, Watari et al. reported improved scoring accuracy following online frame‐of‐reference training compared with untrained controls, although no direct comparison with in‐person training was made.^42^

#### 3.3.2 Online asynchronous delivery

Gormley et al. described an e‐learning intervention focused on awarding global grades followed by scoring at least three video performances, with feedback comparing ratings to previous users.^33^ The intervention increased self‐reported confidence, but effects on scoring reliability or accuracy were not assessed. Moreno‐López and Sinclair similarly reported generally positive examiner perceptions of e‐learning with mixed effects on scoring accuracy.^35^

#### 3.3.3 In‐person delivery

In‐person workshops varied from short presentations to multi‐hour structured sessions involving video scoring, role play, group discussion and standard‐setting exercises.^31^, ^32^, ^34^, ^38^, ^46^ Effects were inconsistent: a 1‐hour workshop using a single exemplar was associated with strong rater agreement for one station,^31^ whereas similar workshop formats demonstrated no statistically significant improvements in reliability in other studies.^32^, ^37^ Participation in formative mock OSCEs was another in‐person strategy to familiarise examiners with scoring requirements. One study reported improved examiner behaviours after a mock OSCE combined with facilitated discussion, compared with untrained participants.^34^ Wilby et al. also incorporated attendance at a formative OSCE within induction, although the effect of this component could not be isolated.^30^

#### 3.3.4 Hybrid approaches

Hybrid approaches combined initial e‐learning with subsequent in‐person workshops.^29^, ^40^ Limited reporting of individual components made attribution difficult, and evaluation was typically confined to participant perceptions or lacked methodological detail.

### 3.4 Temporal influencers of calibration

#### 3.4.1 Timing of case‐orientation calibration to subsequent scoring and durability of effect

Among studies examining case‐orientation calibration, four assessed its impact on examiner scoring immediately following the calibration session,^26^, ^36^, ^37^, ^41^ one at four days,^44^ two after 1 month^42^, ^43^ and one at 2 months.^39^ The remainder did not specify the interval.^29^, ^34^, ^38^ No consistent pattern emerged. Immediate assessments showed mixed effects, and no improvements were observed at 4 days or 2 months. In contrast, the two studies with 1‐month follow‐up reported sustained improvements in scoring reliability and accuracy. One study reported no benefit among raters with high baseline accuracy, suggesting a ceiling effect.^42^

#### 3.4.2 Duration

Interventions ranged from 1‐hour workshops to multi‐day and month‐long programmes, although total training time was often unclear. Available evidence did not show a consistent relationship between duration and improved reliability or accuracy. One study reported progressive improvement in scoring reliability and accuracy across a 3‐day programme,^36^ whereas a month‐long weekly programme showed no statistically significant improvement in reliability.^43^ A 1‐h session was sufficient for most examiners to achieve a target score in one study^31^, but increased marking variability was observed after three 3‐h workshops in another.^38^ No improvement was observed after a 1‐h session^32^ or after 2.5‐h calibration compared with a 30‐min session or no training.^39^ Interview data suggested extended calibration may reduce engagement because of cognitive overload.^41^

## 4 DISCUSSION

This scoping review synthesises the literature on examiner training and calibration for SOCCAs and identifies an evidence base that remains heterogeneous and methodologically inconsistent. Although examiner preparation is widely regarded as essential for ensuring defensible and fair assessment outcomes, the reviewed studies do not demonstrate a clear consensus on best practice for achieving consistent improvements in examiner accuracy or reliability. Moreover, only one study evaluated outcomes beyond scoring metrics to examine the effects on examiner behaviours. Reported effects appear contingent on delivery format, timing, contextual specificity and on assumptions about the nature of performance judgement.

### 4.1 General examiner training and its limitations

General examiner training was rarely the primary focus of the studies reviewed and was more often described as preparatory context for subsequent calibration or scoring activities. As this training is generally intended to promote procedural consistency and clarify examiner roles, many evaluations concentrated on perceived preparedness and self‐reported confidence. This emphasis is not misplaced, given established evidence that confidence and ambiguity influence judgement under conditions of uncertainty.^47^, ^48^ However, it remains unclear whether increased confidence or role clarity translates into greater adherence to expected conduct, responsibilities or scoring standards in practice.

### 4.2 Calibration approaches

A useful distinction identified from this review is between general calibration, which aims to align performance standards in a broad sense, and case‐orientation calibration, which is tailored to the specific station for an upcoming SOCCA. Case orientation is conceptually aligned with case (context) specificity, whereby performance does not reliably generalise across cases, even when similar constructs are assessed.^49^, ^50^ Across both calibration types, frame‐of‐reference training was the predominant strategy employed, despite the availability of alternative rater training strategies.^51^ Viewed through the epistemological distinction introduced earlier, this dominance suggests that much of the literature implicitly treats examiner variability as a problem of psychometric alignment and score convergence, assuming that misaligned internal standards can be corrected through shared exemplars and facilitated discussion, rather than as an interpretive phenomenon requiring shared reasoning and contextual negotiation.

Although our second research question sought to examine how different calibration modalities influence examiner scoring, the near‐exclusive focus of the existing literature on frame‐of‐reference interventions did not permit meaningful comparison between approaches. This reflects a gap in the evidence base rather than evidence of the superiority of any single calibration strategy. While several studies reported modest improvements in scoring reliability or accuracy, effects were inconsistent and, in some cases, accompanied by increased examiner stringency. Alternative approaches to examiner variability have focused on quantifying and adjusting examiner effects rather than attempting to modify them, demonstrating that systematic differences between examiner groups can persist despite standardisation and may require measurement‐based solutions.^52^ Where positive effects were observed, facilitated discussion appeared to be a critical component, consistent with work on assessor cognition indicating that exemplars alone are unlikely to align judgements without explicit articulation and negotiation of reasoning processes.^8^

Notably, the largest and methodologically most robust study included in this review employed a multicentre randomised controlled design to compare VBB case‐orientation calibration with a control group and found no effect on overall examiner variability, although scoring accuracy improved for borderline performances.^45^ Taken together, these findings suggest that calibration may be most useful in areas of highest uncertainty, where examiners experience difficulty discriminating performance near critical decision thresholds.^53^

### 4.3 Station type and scoring approaches

Insufficient methodological detail in the included studies limited systematic categorisation of station competencies and marking formats, and thus constrained inferences about influencers of calibration effectiveness. Nonetheless, there are plausible reasons why station type and scoring approach could shape calibration outcomes. Procedural and physical examination stations often have relatively explicit performance expectations that lend themselves to checklists or detailed analytic rubrics. Such formats may support calibration by constraining the range of acceptable interpretations. By contrast, stations assessing clinically reasoned history taking or communication skills more commonly rely on global judgements and allow multiple defensible approaches, and for which examiner variation may reflect legitimate differences in professional reasoning and weighting of performance dimensions. In these contexts, calibration may be more complex to achieve, requiring shared understandings of quality and adequacy. Related findings support this distinction. For example, Koo et al. reported that examiner training for a student‐delivered peer OSCE improved scoring accuracy for a physical examination station but not for communication‐based stations.^54^ Other work has also found that examiner experience and seniority were associated with differential scoring in communication domains but not in physical examination skills, suggesting that certain station types may be more susceptible to interpretive variability.^55^

### 4.4 Assessor workload and temporal effects

Evidence suggests that elevated cognitive load and temporal dynamics can influence scoring consistency.^47^ Judgements may shift during an OSCE because of fatigue, adaptation or contrast effects, indicating that variability can arise not only from differences between examiners but also from fluctuations within examiners over time.^56^, ^57^ This has practical implications for calibration design. Calibration may be helpful but insufficient if it does not address the conditions under which judgements are made.^9^, ^58^, ^59^, ^60^ Assessors' cognitive load may be reduced through provision of high‐quality written guidance, including explicit marking criteria and up‐to‐date clinical content.^61^ Despite the recognised importance of these contextual factors, no study explicitly reported incorporating exploration of such factors within their training or calibration interventions.

Across the included studies, no consistent pattern was observed between the timing of calibration activities and subsequent examiner scoring outcomes. Importantly, this lack of consistency persisted even among studies that examined scoring immediately or shortly after calibration, a design choice that may reflect an implicit assumption that temporal proximity and recency might support alignment of judgement. The observed inconsistency suggests that recency alone cannot be assumed to account for calibration effectiveness. Instead, other factors, such as the nature of the calibration activity, the quality of facilitated discussion and examiner engagement, may be more influential than timing per se.

### 4.5 Epistemological tensions in performance‐based assessment

Insights from assessor cognition research offer a valuable framework for understanding examiner variation. Variability may arise from differences in cue salience, uncertainty about criteria and idiosyncratic integration of information.^7^ Examiners also draw on embedded personal theories of competence shaped by clinical experience and professional socialisation.^62^ Yet many calibration interventions adopt an implicitly positivist orientation, presuming a ‘true’ score and positioning calibration as a mechanism to reduce construct‐irrelevant variance and improve classification accuracy. Sociocultural and constructivist perspectives, by contrast, conceptualise judgements as interpretive, situated and shaped by professional identity and context.^8^, ^59^, ^63^

These differing assumptions raise fundamental questions about the purpose of calibration: whether its aim should be convergence on uniform scores or the development of defensible reasoning processes. Increased stringency following calibration may reflect closer alignment with a reference standard, but it may also reflect suppression of legitimate professional judgement. The reviewed studies did not engage explicitly with these theoretical assumptions, limiting both conceptual coherence and interpretive clarity.

Beyond these conceptual tensions, calibration practices also carry important implications for fairness and bias in high‐stakes assessment. Although equity, diversity and inclusion were not the focus of this review, calibration necessarily intersects with issues of fairness and bias in high‐stakes assessment, particularly where judgments are made under conditions of uncertainty or at decision thresholds.

Importantly, the epistemological tensions identified in this review are not unique to simulation‐based assessments. Recent conceptual work examining rater training within workplace‐based assessment has similarly questioned the coherence of traditional psychometric aims, such as accuracy, reliability and error reduction, with contemporary understandings of assessment as situated, interpretive and socially mediated.^64^ Although SOCCAs differ structurally from WBAs, the patterns observed in this review mirror many of the limitations highlighted in broader rater‐training scholarship, including inconsistent effects, continued reliance on frame‐of‐reference approaches and limited attention to how examiner judgement is formed and negotiated. This convergence suggests that the challenges associated with examiner training and calibration reflect more fundamental epistemological tensions within assessment practice, rather than issues specific to any single assessment format.

### 4.6 Recommendations for future research

Future studies should more clearly disentangle the effects of general training, general calibration and case‐orientation calibration, specifying the intended mechanisms and selecting outcome measures that align with those approaches. Evaluation should extend beyond the rater metrics of reliability and accuracy to include examiner behaviour and decision‐making, such as adherence to marking guidance, interactional conduct within stations and judgements under uncertainty. In particular, further work should examine how examiner experience and seniority influence responses to training and calibration, and whether these factors shape behavioural as well as scoring outcomes. Key design parameters (including exemplar number and quality, performance levels represented, the role of facilitated discussion and the timing of interventions relative to live examining) remain underexplored. Research exploring approaches beyond frame‐of‐reference would also clarify the relative effectiveness of alternative strategies. The optimal timing and proximity of training and calibration delivery before live examining, the durability of calibration effects over time and the value of refresher sessions across examination cycles are important areas for further work. Additionally, studies could investigate if and how station competency and marking rubric type influence calibration outcomes. Building on this mapping of the literature, future research could usefully incorporate stakeholder consultation to explore how examiner training and calibration practices are designed, experienced and implemented in practice, and to inform the development of contextually responsive interventions.

### 4.7 Limitations of this review

The heterogeneity of study designs, interventions, outcomes and contexts constrained synthesis to descriptive comparison and limited conclusions about comparative effectiveness. Variation in terminology and operational definitions of training and calibration required interpretive judgements during categorisation and may have contributed to misclassification. EMBASE was not searched as part of this review, which may have resulted in the exclusion of some relevant European or allied health literature not fully indexed in the databases searched. Consistent with scoping review conventions, no formal quality appraisal was undertaken; consequently, the review maps the characteristics and extent of the literature but cannot determine the robustness of individual effects. As such, it is not possible to determine whether mixed or null findings reflect true ineffectiveness of examiner training and calibration interventions or methodological limitations within the primary studies. Publication bias is possible, and restriction to English‐language studies may have excluded relevant evidence.

## 5 CONCLUSIONS

Despite widespread endorsement of examiner training and calibration within structured clinical assessments by regulators and professional bodies, the empirical foundation supporting these recommendations remains limited and inconsistent. Given the resource implications and high‐stakes consequences of these assessments, the routine incorporation of these interventions warrants stronger empirical justification. Without such evidence, there is a risk that they become ritualistic: implemented because they are expected and intuitively persuasive, rather than on the basis of a robust and well‐articulated empirical rationale.

## AUTHOR CONTRIBUTIONS

**Harish Thampy:** Conceptualization; writing—original draft; methodology; validation; writing—review and editing; supervision; data curation; project administration; formal analysis. **Niamh Callanan:** Investigation; writing—original draft; methodology; writing—review and editing; data curation; formal analysis; project administration. **Arslan Ahmed:** Investigation; writing—original draft; methodology; writing—review and editing; data curation; formal analysis; project administration. **Sophia Taromsari:** Investigation; writing—original draft; methodology; writing—review and editing; data curation; formal analysis; project administration.

## CONFLICT OF INTEREST STATEMENT

The authors do not have any conflicts of interest to declare.

## ETHICS STATEMENT

This research does not involve the use of human subjects. Therefore, ethical approval was not required for this study.

## Data Availability

The data that support the findings of this study are available from the corresponding author upon reasonable request.
